# High-throughput sequencing reveals the presence of novel and known viruses in diseased *Paris yunnanensis*

**DOI:** 10.3389/fmicb.2022.1045750

**Published:** 2022-12-14

**Authors:** Ping-xiu Lan, Peng He, Jie Yang, Guo-hua Zhou, Xiao-jiao Chen, Tai-yun Wei, Chen-rong Li, Rong Gu, Ruhui Li, Fan Li

**Affiliations:** ^1^State Key Laboratory for Conservation and Utilization of Bio-Resources in Yunnan, Yunnan Agricultural University, Kunming, China; ^2^Yunnan Baiyao Group, Chinese Medicine Resources Co., Ltd., Kunming, China; ^3^State Key Laboratory of Ecological Pest Control for Fujian and Taiwan Crops, Fujian Agriculture and Forestry University, Fuzhou, China; ^4^USDA-ARS, National Germplasm Resources Laboratory, Beltsville, MD, United States

**Keywords:** *Paris yunnanensis*, high-throughput sequencing, virome, Paris potyvirus 3, Paris potyvirus 4, Paris nepovirus 1, distribution

## Abstract

*Paris* spp. are important medicinal plant and main raw material for many Chinese patent medicines, but viral diseases have became serious problems in cultivation of this group of important medicinal plants in China. In this study, eight viruses were identified in the diseased plants of *Paris yunnanensis* by high-throughput sequencing (HTS) and RT–PCR. These viruses include three novel viruses (two potyviruses and one nepovirus), Hippeastrum chlorotic ringspot virus (HCRV), Lychnis mottle virus (LycMoV), Paris mosaic necrosis virus (PMNV), Paris virus 1 and pepper mild mottle virus. The three new viruses were tentatively named Paris potyvirus 3 (ParPV-3), Paris potyvirus 4 (ParPV-4), Paris nepovirus 1 (ParNV-1) and their complete genome sequences were determined. Sequence analyses showed ParPV-3 and ParPV-4 shared the highest amino acid (aa) sequence identities of 54.3% to each other and 53.0–57.8% to other known potyviruses. ParNV-1 had aa sequence identities of 28.8–63.7% at protease-polymerase (Pro-Pol) with other nepoviruses. Phylogenetic analyses further support that the three viruses are new members of their corresponding genera. Analyses of the partial sequences of HCRV and LycMoV infecting *P. yunnanensis* revealed they diverged from existing isolates by aa sequence identities of 97.1% at glycoprotein precursor of HCRV and 93.3% at polyprotein of LycMoV. These two viruses are reported for the first time in *Paris* spp. A total of 123 field samples collected from *P. yunnanensis* in four counties of Yunnan, Southwest China were tested by RT–PCR for detecting each of the eight viruses. Results showed that nearly half of the samples were positive for at least one of the eight viruses. Two potyviruses, ParPV-3 (26.8%) and PMNV (24.4%), were predominant and widely distributed in the fields, while other viruses occurred in low rates and/or had limited distribution. This study insights into the virome infecting *P. yunnanensis* and provides valuable information for diagnosis and control of viral diseases in *P. yunnanensis.*

## Introduction

*Paris* (chonglu in Chinese) is a genus of perennial rhizomatous plants in the family Melanthiaceae that is widely distributed in Asia and Europe, with a center of diversity in China (Cunninghama et al., 2018; Ji, 2021). The herbaceous plants are usually grown in clumps in humid and shady forests at high altitudes. The genus contains at least 26 species, and most of them are used for treating diseases such as bone fractures, traumatic injuries, snakebite, mumps and abscess in traditional medicine (Jing et al., 2017; Ji, 2021). Modern pharmacological studies have proved that *Paris* spp. contain steroidal saponins, plant ecdysteroids, phytosterols, flavonoids and other phytochemicals with anti-tumor, anti-diabetic, antioxidant, anti-inflammatory and anti-fungal activities (Mastuda et al., 2013). At least 81 patented Chinese medicines including well-known Yunnan Baiyao and snakebite medicine use the dried rhizome of *Paris* spp. as an important ingredient (Tao et al., 2020).

Sources of the rhizomes of *Paris* spp. are usually wild plants. Demand for the rhizomes has been increasing since the beginning of this century and reached to 3,000 tons that the wild populations cannot sustainably supply (Li et al., 2015). Nineteen species of the genus are currently listed as endangered due to overharvesting (Tao et al., 2020). Domestication and cultivation of wild *Paris* spp. is an effective way to solve the market demand and protect wild resources of *Paris* spp. Planting of *Paris* spp. started as early as 1980s and has been increased rapidly in past 20 years (Ji, 2021). The annual planting area is now more than 10,000 hm^2^ with a production of 50,000 tons and market value of 10 billion CNY (*ca.* 1.6 billion USD) in China.

*Paris yunnanensis* (Dian chonglou in Chinese) is one of the two *Paris* species included in the Chinese Pharmacopoeia (2015 Edition I). Yunnan is the origin and main production province of *P. yunnanensis*, accounting for 78% of the total planting area and 60% of the production in China. However, cultivated plants are prone to infection and accumulation of viruses due to high-density planting in the fields and long growth period of 7–10 years. Six viruses, Paris polyphylla virus X (PPVX, genus *Potexvirus*, family *Betaflexiviridae*) (Dong et al., 2007), Paris mosaic necrosis virus (PMNV, genus *Potyvirus*, Family *Potyviridae*) (Lan et al., 2018), Paris virus 1 (ParV1, genus *Potyvirus*) (Chen et al., 2020), chilli veinal mottle virus (ChiVMV, genus *Potyvirus*) (Yang et al., 2022), pepper mild mottle virus (PMMoV, genus *Tobamovirus*, family *Virgaviridae*) (Wen et al., 2019) and Paris virus 2 (ParV2, genus *Tobamovirus*) (Chen et al., 2021), have recently been reported to infect and cause diseases of foliar mottle, necrotic, mosaic, chlorosis and plant stunting in *P. yunnanensis*.

In 2017, a viral disease outbreak occurred in the fields of *P. yunnanensis* in Mangshi, Yunnan province, Southwest China. Leaf samples of 9 plants with severe chlorosis (yellowing) were brought to our attention by local extension specialists. The samples tested positive for PMNV (Lan et al., 2018). To further investigate the causal agents of different viral diseases in *P. yunnanensis*, surveys were conducted in 2019 in four counties, Lijiang, Lushui, Mangshi and Tengchong, where additional 114 symptomatic samples were collected. Transmission electron microscopy (TEM) and high-throughput sequencing (HTS) were performed on two pooled samples, one from the 9 Mangshi samples of 2017 and another from 11 samples collected in Lijiang in 2019. Two types of viral morphology were observed under TEM. Seven viruses, two new potyviruses, one new nepovirus and four known viruses [Lychnis mottle virus (LycMoV), Hippeastrum chlorotic ringspot virus (HCRV), PMNV and ParV1], were identified by HTS analysis. RT-PCR using virus-specific primers were used to detect the Paris viruses in 123 samples, and seven viruses identified by HTS in this study and PMMoV were detected in the diseased plants of *P. yunnanensis*. The occurrences of these viruses in the fields were assessed.

## Materials and methods

### Symptoms and sampling

**Figure 1:**
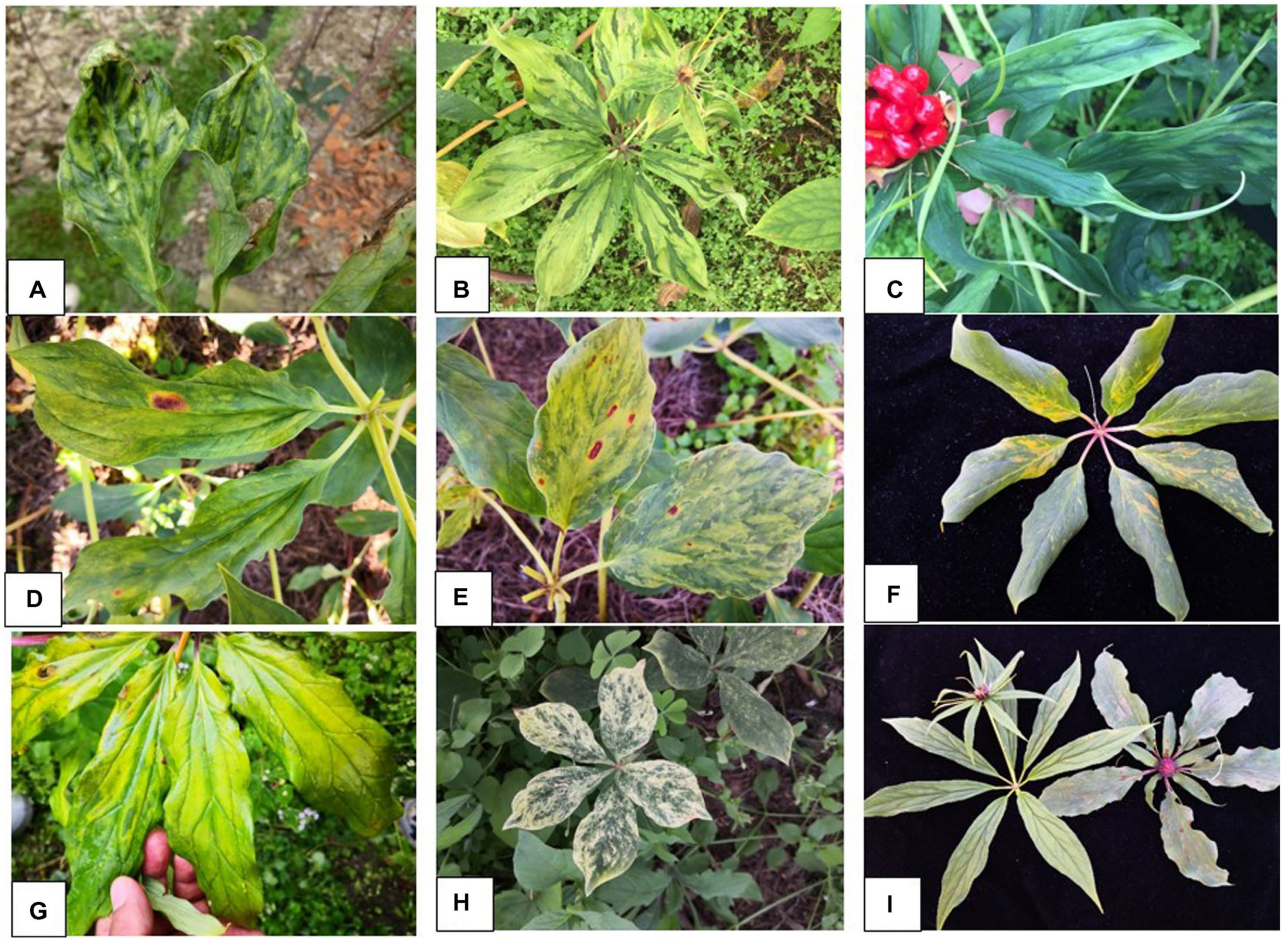
Symptoms of virus diseases on leaves of *Paris yunnanensis* plants: **(A)** mosaic and deformation, **(B)** mosaic and green banding, **(C)** mottle and linear leaf, **(D)** chlorosis and necrosis, **(E)** mosaic and necrosis, **(F)** macula and chlorosis, **(G)** mosaic and chlorosis, **(H)** whiting, and **(I)** pedicel austerity.

During 2017 and 2019, virus-like symptoms were observed in farms of *P. yunnanensis* in four counties [Mangshi (August of 2017 and 2019); Lijiang and Tengchong (October 2019); Lushui (April 2019)] in Northwest Yunnan. The symptoms included foliar mosaic and deformation (Figure 1A), mosaic and green banding (Figure 1B), mottle and linear leaf (Figure 1C), chlorosis and necrosis (Figure 1D), mosaic and necrosis (Figure 1E), macula and chlorosis (Figure 1F), mosaic and chlorosis (Figure 1G), whiting (Figure 1H), pedicel austerity (Figure 1I). The symptoms were more severe in Mangshi than other three counties. The Mangshi samples were collected from two different farms in 2017 and 2019. Severe yellowing symptom were observed in 2017, while foliar chlorosis, mosaic, deformation and plant stunt were found in 2019. Twenty leaf samples of various symptoms collected from Mangshi (9 samples in 2017) and Lijiang (11 samples in 2019) were pooled into two samples labelled as Mshi-CL and Lji-CL, respectively. The two samples were frozen in liquid nitrogen and then shipped on dry ice to Origingene Bio-Pharm Technology Co. (Shanghai, China) for HTS. Additional 103 leaf samples were also collected from the four counties in 2019 and stored at −20°C until use. Except one sample, all other samples were collected from symptomatic plants.

### Transmission electron microscopy

Leaf tissues of the two pooled samples (Mshi-CL and Lji-CL) were ground into fine powder in liquid nitrogen. The powder of 0.1 g was transferred to a 1.5-ml microcentrifuge tube containing 300 μl of 200 mM sodium phosphate (NaPO4) buffer (pH 7.4, containing 2.5% glutaraldehyde). After a centrifugation at 12,000 rpm for 10 min (4°C), a drop of the supernatant was placed on a piece of sealing film. A carbon-coated grid was then placed upside down on the top of the drop for 2–3 min. The excess liquid on the grid was removed by a filter paper, and the grid was stained in a drop of 2% phosphotungstic acid (pH 6.8) for 2 min and observed under a transmission electron microscope. Images with virus particles were digitally recorded and some of them were measured.

### High-throughput sequencing and data analyses

Total RNAs were isolated from leaf tissues of the two pooled samples (Mshi-CL and Lji-CL) using Trizol reagent (Invitrogen, Carlsbad, CA, USA) following the manufacturer’s instructions, respectively. The RNA purity, concentration and integrity were determined by NanoDrop 2000 (NanoDrop, Wilmington, DE, USA) and Agilent 2,100 until OD260/280 equal to 1.6 ~ 1.8. Ribosomal RNAs were depleted using a TruSeq RNA Sample Prep Kit, and the remaining RNAs were used for construction of RNA-seq libraries, which were sequenced on an Illumina HiSeq X-ten platform with paired-end reads length of 150 bp. After removal of failed reads, the qualified RNA reads were assembled *de novo* by CLC Genomics Workbench 20.1 (Qiagen, Germantown, MD, USA). The obtained contigs were annotated by Blast searches against local Virus_NR and viroid datasets retrieved from the National Center for Biotechnology Information (NCBI) GenBank.

### Validation of viruses

Total RNAs were extracted from each of the 20 HTS samples using EasyPure Plant RNA kit (TransGen Biotech, Beijing, China) according to the manufacturer’s instructions. Presence of both novel and known viruses identified by HTS was confirmed by RT-PCR using virus-specific primers (Supplementary Table 1) and subsequent Sanger sequencing of the amplicon clones. The complete genomic sequences of the three new viruses were obtained from sequence assemblies of the amplicons generated from RT-PCR and 5’ RACE reactions, respectively. The partial genomic sequences of the LycMoV and HCRV isolates were assembled from sequences of the RT-PCR amplicons, respectively. Virus-specific primers were designed according to the contig sequences of each of the target viruses (new or new-host) obtained in this study (Supplementary Tables 1–5). All neighboring primers have an overlapping region of 115–151 nt at the ends of the contiguous amplicons (Supplementary Tables 2–5). RT-PCR was conducted using a PrimeScriptTM One-step RT-PCR Kit ver. 2 (TaKaRa Biotechnology Co. Ltd., Dalian, China). The 10 μl RT-PCR reaction contained 1 μl of total RNAs, 0.5 μl of each primer (5 μM), 5 μl of 2× Reaction Mix, 0.4 μl of Enzyme Mix and 2.6 μl of distilled water. The thermal cycling conditions were 1 cycle of reverse transcription at 50°C for 30 min and denaturation at 94°C for 2 min, 35 cycles of amplification at 94°C for 30 s, 55°C for 30 s and 72°C for 90 s, and a final extension of 72°C for 10 min. The amplicons were resolved in 1% agarose gels in TAE buffer, and the bands with expected size were excised, gel-purified and cloned into pMD19-T vector (TaKaRa Biotechnology). The overnight cultures of at least three clones were sequenced for each amplicon (BGI, Guangzhou, China).

The 5′-terminal sequences of the novel viruses were amplified using SMARTer®RACE 5′/3′ kit (TaKaRa Biotechnology) according to the manufacturer’s instructions. Their 3′-terminal sequences were obtained from cDNA synthesis using an oligo (dT)-type primer Viral8, followed by PCR using a virus-specific forward primer and the degenerate primer of viral9 (Supplementary Tables 2–4). The cDNA synthesis was conducted using M-MLV reverse transcriptase according to the manufacturer’s instructions (TaKaRa Biotechnology). PCR was carried out using GreenTaq Mix (Vazyme Biotech Co., Ltd., Nanjing, China) under the thermal cycling conditions as follows: pre-denaturation at 94°C for 2 min, 35 cycles of 94°C for 30 s, 55–60°C for 30 s and 72°C for 1 min, and a final extension of 72°C for 10 min.

### Genome and phylogenetic analysis

The genomic sequences of the viruses were assembled from sequences of the RT-PCR and RACE amplicons using DNASTAR 7.0 package (DNASTAR Inc., Madison, WI, USA). Open reading frames (ORFs) were predicted using ORF finder at.^1^ Pairwise comparisons of nucleotide and amino acid sequences were performed using the Pairwise Sequence Alignment of EMBOSS Needle at.^2^ Multiple sequence alignment was conducted using Clustal Omega at.^3^ Nine highly conserved proteolytic cleavage sites in the polyprotein of the new potyviruses were predicted using a multiple alignment of relevant potyviruses polyprotein and the criteria proposed by Adams et al. (2005a). Putative dipeptides of cleavage sites in the two polyproteins of new nepovirus were deduced based on the alignment of members of subgroup B of the genus *Nepovirus*. Conserved domains of the nepovirus were predicted using the conserved Domain Search at.^4^ Phylogenetic trees were constructed using maximum likelihood (ML) method in the MEGA7 (Kumar et al., 2016).

### Virus detection and field survey

RT-PCR was performed as described above. Total nucleic acids were isolated from leaves of the samples using a CTAB-based method (Li et al., 2008) for determination of infection rate of each of ten viruses infecting *Paris* spp. These viruses included seven viruses (ParPV-3, ParPV-4, ParNV-1, HCRV, LycMoV, PMNV, and ParV1) identified by HTS in this study and three viruses (ChiVMV, PMMoV and PPVX) that were not found in this study but reported previously (Wen et al., 2019; Chen et al., 2021; Yang et al., 2022). All the samples (20 HTS and 103 additional samples) collected from the four counties were tested individually for each of the target viruses. Virus-specific primers (Supplementary Table 1) were designed according to the genomic sequence obtained from this and previous studies. RT-PCR was performed as described above. RT-PCR products were separated on 1.0% agarose gels by electrophoresis and visualized by Gel Imaging System (GenoSens 1800, Clinx Science Instruments Co., Ltd., ShangHai, China).

## Results

### Virus particles

**Figure 2:**
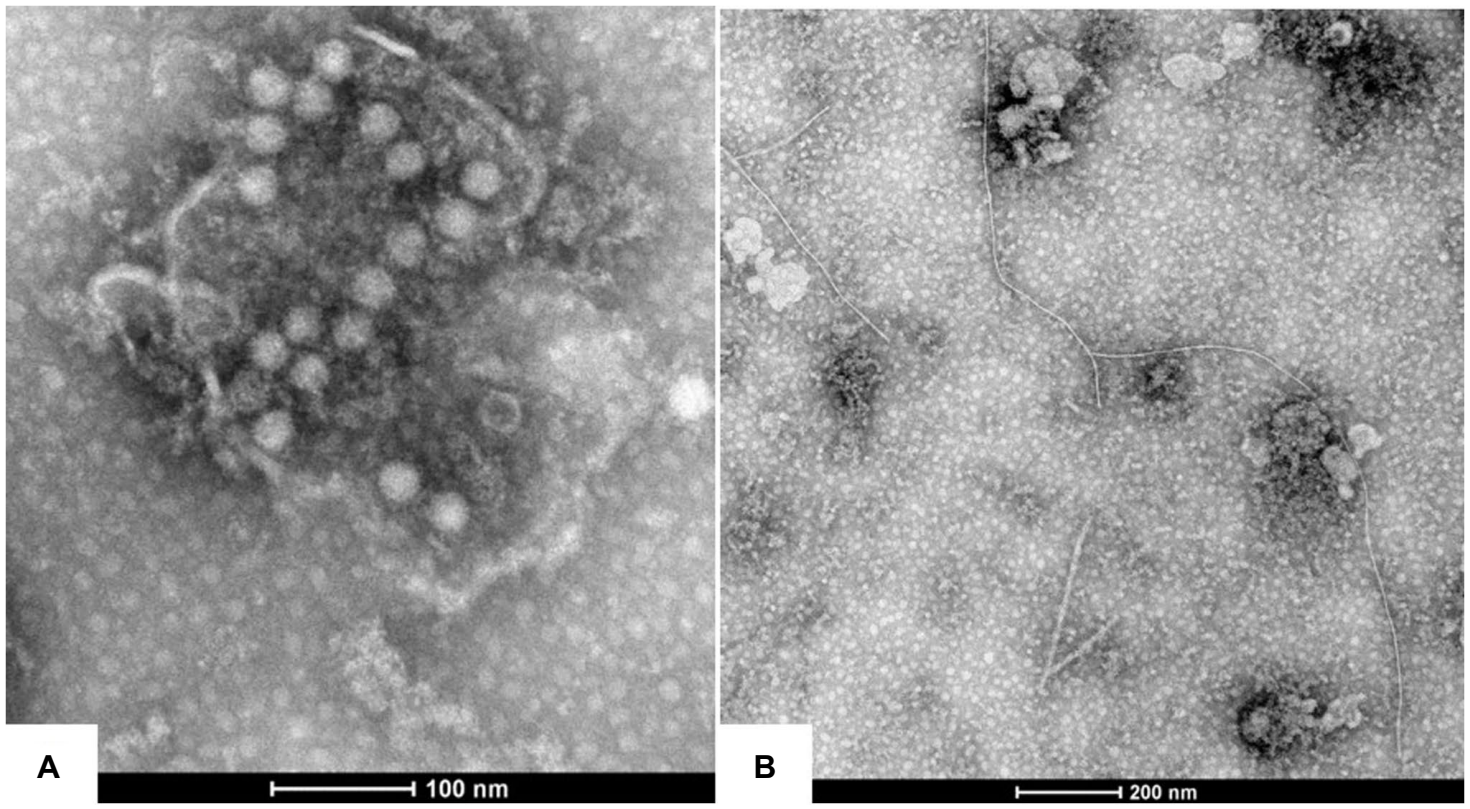
Virions morphology associated with the virus diseases of *P. yunnanensis*: **(A)** spherical virion with a diameter of about 25 nm, **(B)** filamentous particles of 700 ~ 800 nm in length.

Two types of viral virions, spherical and filamentous shapes, were observed under TEM in the leaf extracts of the symptomatic *P*. *yunnanensis* samples. The spherical particles were measured to be 25–30 nm in diameter (Figure 2A), and filamentous particles were 700 ~ 800 nm in length (Figure 2B). The results indicated that viruses with spherical shape, besides viruses with filamentous shape (Dong et al., 2007; Lan et al., 2018; Wen et al., 2019; Chen et al., 2020, 2021; Yang et al., 2022), were associated with the viral diseases of *P. yunnanensis*.

### Identification of viruses in *Paris yunnanensis* by high-throughput sequence

HTS of the two pooled samples, Mshi-CL and Lji-CL, yielded 53,532,438 and 50,522,768 raw reads, respectively. A total of 301,834 and 179,957 contigs [≥ 200 nucleotides (nt)] were assembled *de novo* from Mshi-CL and Lji-CL, respectively. BLASTx search revealed the presence of large contigs with the highest amino acid (aa) sequence identities to four viruses [Kalanchoe mosaic virus (KMV, genus *Potyvirus*, family *Potyviridae*; 52–57%), Hippeastrum chlorotic ringspot virus (HCRV, genus *Orthotospovirus*, family *Tospoviridae*; 97%), Lychnis mottle virus (LycMoV, genus *Stralarivirus*, family *Secoviridae*; 95%) and PMNV (93–100%)] and four viruses [grapevine Anatolian ringspot virus (GARSV, genus *Nepovirus,* family *Secoviridae*; 55% of RNA1 and 39% of RNA2), iris potyvirus A (IPVA, genus *Potyvirus*, family *Potyviridae*; 57%), PMNV (88–93%), ParV1 (89%)] in Mshi-CL and Lji-CL, respectively (Supplementary Table 6). Two large contigs of the IPVA-like potyvirus (82% nt identity between them) were found in Lji-CL, while two large contig of KMV-like potyvirus (91% nt identity between them) were found in Mshi-CL. Several large contigs of PMNV were found in Lji-CL (4 contigs with 80–87% nt identities) and Mhi-CL (3 contigs with 85–86% nt identities), respectively. These contigs represented nearly full-length genomic sequences of the corresponding viruses. Multiple contigs of PMNV were also present Mshi-CL (155 contigs in a range of 311–7,514 nt) and Lji-CL (148 contigs in a range of 224–6,288 nt), respectively. The average coverage of PMNV were 60,056x (7.8% total reads) in Mshi-CL and 137,233x (16.7% total reads) in Lji-CL. The average coverages were 20,425x for the KMV-like potyvirus, 2,782x of RNA1 and 2,242x of RNA2 for the nepovirus, 383x for IPVA-like potyvirus, 30x for HCRV, 25x for LycMoV, and 4,159x for ParPV-1. Infections of these viruses were further confirmed by RT-PCR using virus-specific primers (Supplementary Table 1). The aa sequence identity values between the IPVA-like, KMV-like or the new nepovirus and its relevant virus were below the species criterion (<80% for both genera) (Adams et al., 2005b; Thompson et al., 2017), suggesting each of them should be new member of the corresponding genus. Thus, the two potyviruses were tentatively named Paris potyvirus 3 (ParPV-3) and Paris potyvirus 4 (ParPV-4), and the new nepovirus was named as Paris nepovirus 1 (ParNV-1), respectively. LycMoV and HCRV were the first report in *P. yunnanensis*, while PMNV and ParV1 were previously reported (Lan et al., 2018; Chen et al., 2020).

### Complete genomic sequences of two novel potyviruses

**Figure 3:**
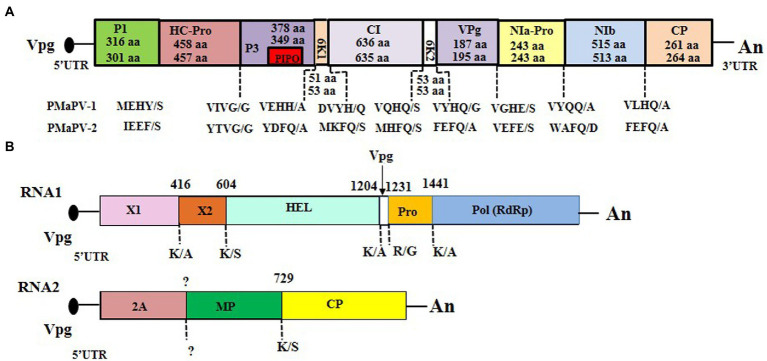
Genome organization of the three novel viruses: **(A)** Paris potyvirus 3 (ParPV-3) (above) and ParPV-4 (below), **(B)** Paris nepovirus 1 (ParNV-1). The 5′- and 3′-untranslated regions (UTR) are represented by a solid line, and the open reading frame (ORF) is depicted by an open box with solid line. The putative protein of PIPO within the P3 of ParPV-3 and ParPV-4 is indicated with a small box in red. The putative proteinase cleavage sites in the polyprotein of the three viruses were labeled below the genome. The size of each protein in ParPV-3 and ParPV-4 are indicated whereas the numbers above the genome of ParNV-1 indicate the start for each region.

The complete genomic sequences of two novel potyviruses were obtained from sequence assembly of the amplicons generated from RT-PCR and 5’ RACE reactions. The complete genomic sequences of ParPV-3 (YMS isolate; GenBank accession number: OK073904) and ParPV-4 (YLJ isolate; accession number: OP374157) were determined to be 9,600 and 9,509 nt, respectively, excluding the 3′-terminal poly(A) tail. The genome organization of ParPV-3 and ParPV-4 are typical of those of members of the genus *Potyvirus* in the family *Potyviridae*, containing a large ORF. ParPV-3 encodes a polyprotein of 3,098 aa residues, and ParPV-4 encodes a polyprotein of 3,063 aa residues. The 5′ untranslated region (UTR) is 155 nt for ParPV-3 and 101 nt for ParPV-4, and 3’ UTRs is 259 nt for ParPV-3 and 219 nt for ParPV-4. Nine highly conserved proteolytic cleavage sites in the polyprotein were identified by comparison with the consensus protease recognition motifs of selected potyviruses, which resulting in ten putative mature proteins of P1 (316 aa for ParPV-3, 301 aa for ParPV-4), HC-Pro (458 aa for ParPV-3, 457 aa for ParPV-4), P3 (378 aa for ParPV-3, 349 aa for ParPV-4), 6 K1 (51 aa for ParPV-3, 53 aa for ParPV-4), CI (636 aa for ParPV-3, 635 aa for ParPV-4), 6 K2 (53 aa for both viruses), VPg (187 aa for ParPV-3, 195 aa for ParPV-4), NIa-Pro (243 aa for both viruses), NIb (515 aa for ParPV-3, 513 aa for ParPV-4) and CP (261 aa for ParPV-3, 264 aa for ParPV-4) (Figure 3A). The small ORF PIPO within the P3 of potyviruses was also identified by the presence of GA_6_ in ParPV-3 (nt 3,007-3,014) and ParPV-4 (nt 2,838–2,844), respectively.

Comparisons of the polyprotein sequences of the two new viruses and other known potyviruses revealed the presence of most conserved motifs (Worrall et al., 2019). These motifs include P1 motif of I-X-F-G (I-T-F-G in ParPV-3; I-I-F-G in ParPV-4) that is associated with protease activity; HC-Pro motifs of C-X_8_-C-X_18_-C-X_2_-C related to aphid transmission, F-R-N-K-X_12_-C-D-N-Q-L-D for symptomatology, H-A-K-R-F-F for cell-to-cell movement and C-X_72−_H for protease activity; CI motifs of D-E-C-H, K-V-S-A-T-P-P, L-V-Y-V, V-A-T-N-I-I-E-N-G-V-T-L and G-E-R-I-Q-R-L-G-R-V-G-R that are associated with the potential helicase activity; NIa-Pro motif of H-X_34_-D-X_67_-G-X-C-G-X_14_-H which is related to proteolytic activity; and NIb motifs of F-T-A-A-P-I-D, C-V-D-D-F-N, G-N-N-S-G-Q-P-S-T-V-V-D-N-S-L-M-V and G-D-D involved in RNA-dependent RNA polymerase activities. Three conserved motifs for aphid transmission activity, “R-I-T-C” at the N-terminus of HC-Pro, “P–T-K” at the C-terminus of HC-Pro and “D-A-G” (ParPV-3) or “N-A-G” (ParPV-4) at the N terminus of the CP (Atreya et al., 1991), are all present in their corresponding proteins. Several reports have showed that “K-I-T-C” replaced by “R-I-T-C” (Atreya and Pirone, 1993), or “D-A-G” replaced by “N-A-G” (Atreya et al., 1991) did not affect the aphid transmission activity.

Pairwise comparisons of the complete genomic sequences of ParPV-3, ParPV-4 and selected members of the genus *Potyvirus* showed that they shared the genomic sequence identities of 54.3% with each other and 53.0–57.8% with other potyviruses. The polyprotein sequence identities are 48.0% between ParPV-3 and ParPV-4 and 39.3–51.9% between them and other potyviruses. These values are all under the current species demarcation criteria (<76% at nt level and < 80% at aa level) for the family *Potyviridae* (Adams et al., 2005b), confirming the two potyviruses are novel members of the genus *Potyvirus*.

**Figure 4:**
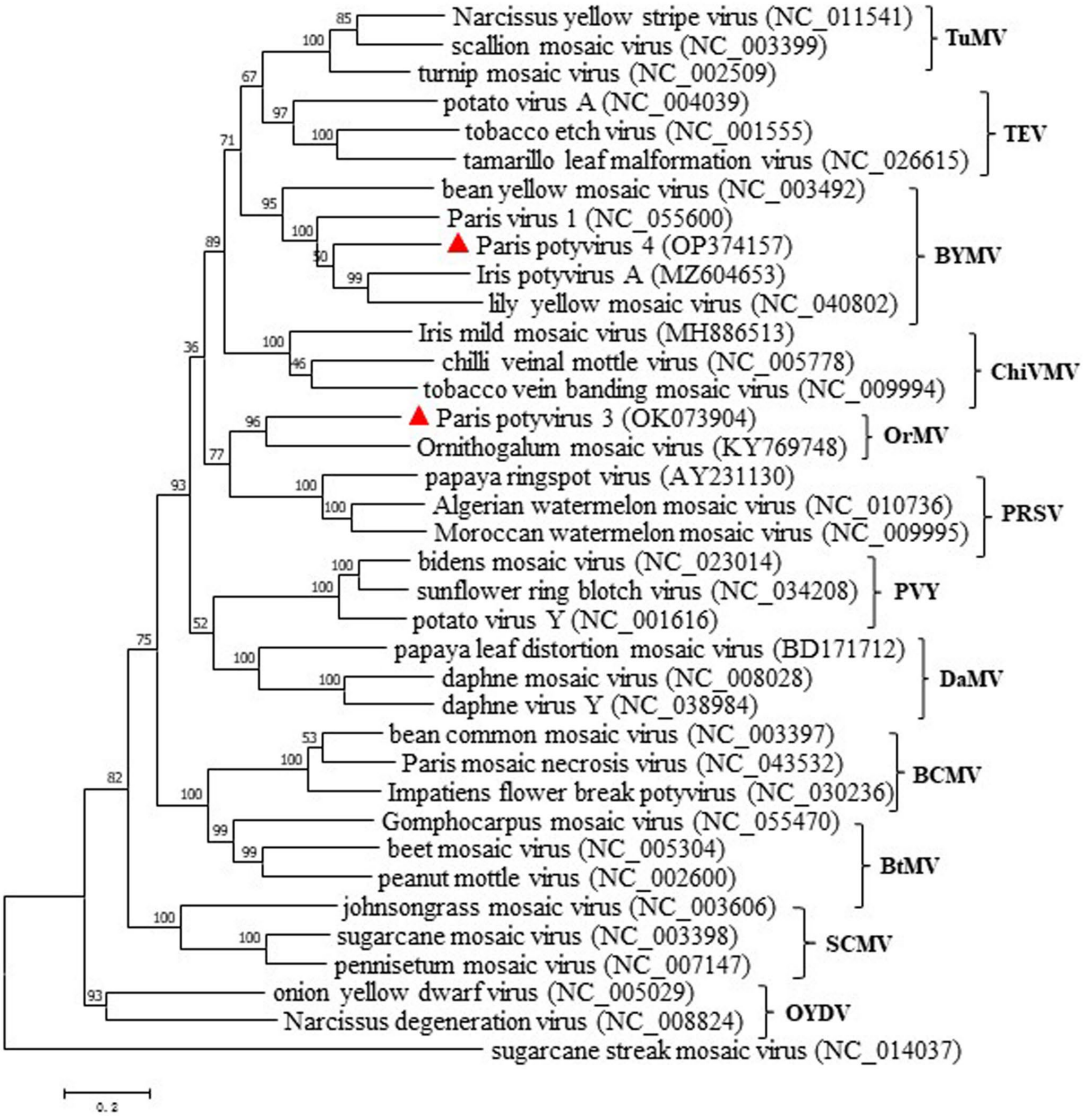
Maximum-likelihood tree based on the deduced polyprotein sequences of ParPV-3, ParPV-4 and representative members of the genus *Potyvirus*. Each of the subgroups is indicated by abbreviation of the representative virus. Bootstrap analysis was applied using 1,000 bootstrap replicates. The scale bar representing a genetic distance of 0.2. Sugarcane streak mosaic virus, a member of the genus *Poacevirus*, was used as an outgroup.

Phylogenetic analyses were first conducted using the deduced polyprotein sequences of ParPV-3, ParPV-4 and other potyviruses available in GenBank, and then simplified to contain them and 36 selected potyviruses (Figure 4). Maximum likelihood tree placed ParPV-3 and ParPV-4 into different subgroups of the genus *Potyvirus*. ParPV-3 was clustered with Ornithogalum mosaic virus (OrMV, KY769748) in a clade (tentatively labeled as OrMV subgroup), while ParPV-4 was grouped together with bean yellow mosaic virus (BYMV, NC_003492), lily yellow mosaic virus (LYMV, NC_040802), IPVA (MA604653), and ParV1 (NC_055600) into the BYMV subgroup. These results supported that Paris potyvirus 3 and Paris potyvirus 4 were two distinct members of genus *Potyvirus*.

### Sequence analyses of a novel nepovirus

Nepoviruses are a group of bipartite viruses with two single-stranded RNAs, each encoding a single polyprotein (Thompson et al., 2017). The complete RNA1 sequence of ParNV-1 was obtained by assembly of the amplicon sequences obtained from the RT-PCR and 5’RACE, but very end of the 5’-UTR of RNA2 was not obtained despite of several 5’ RACE reactions. Approximately 56 nt of RNA2 were missed according to the comparisons with several closely related nepoviruses. RNA1 (YLJ isolate; GenBank accession number: OP374158) and RNA2 (YLJ isolate; GenBank accession number: OP374159) of ParNV-1 are 7,324 nt and 4,604 nt long, respectively, excluding a poly(A) tail (Figure 3B). The 5’-UTR is 253 nt for RNA1 and 228 nt (not complete) for RNA2, and the 3’-UTR is 243 nt for RNA1 and 245 nt for RNA2. Like other members of the genus *Nepovirus*, the terminal sequences of the two ParNV-1 RNAs also have high sequence identities. The identities are 77.4% for the 5’-UTRs and 96.1% for the 3’-UTRs between the two RNAs of ParNV-1.

The genomic organization of ParNV-1 is very similar to those of other members of the genus *Nepovirus* (Figure 3B). RNA1 is predicted to encode a polyprotein (P1) of 2,276 aa residues (254.6 kDa) with conserved domains of RNA helicase (HEL) (aa 775–876) and RNA-dependent RNA polymerase (RdRp) (aa 1,629-1,914). The polyprotein of RNA2 (P2) is 1,377 aa (152.6 kDa) consisting of a putative movement (MP) and capsid protein (CP). The cleavage sites of each polyprotein were predicted using MUSCLE alignments of polyprotein sequences of the nepovirus subgroup B viruses to be K_416_/A_417_ (X1/X2), K_604_/S_605_ (X2/NTB), K_1204_/A_1205_ (NTB/VPg) and K_1441_/A_1442_ (VPg/Pol) in P1, and K_729_/S_730_ (MP/CP) in P2 (Figure 3B).

**Figure 5:**
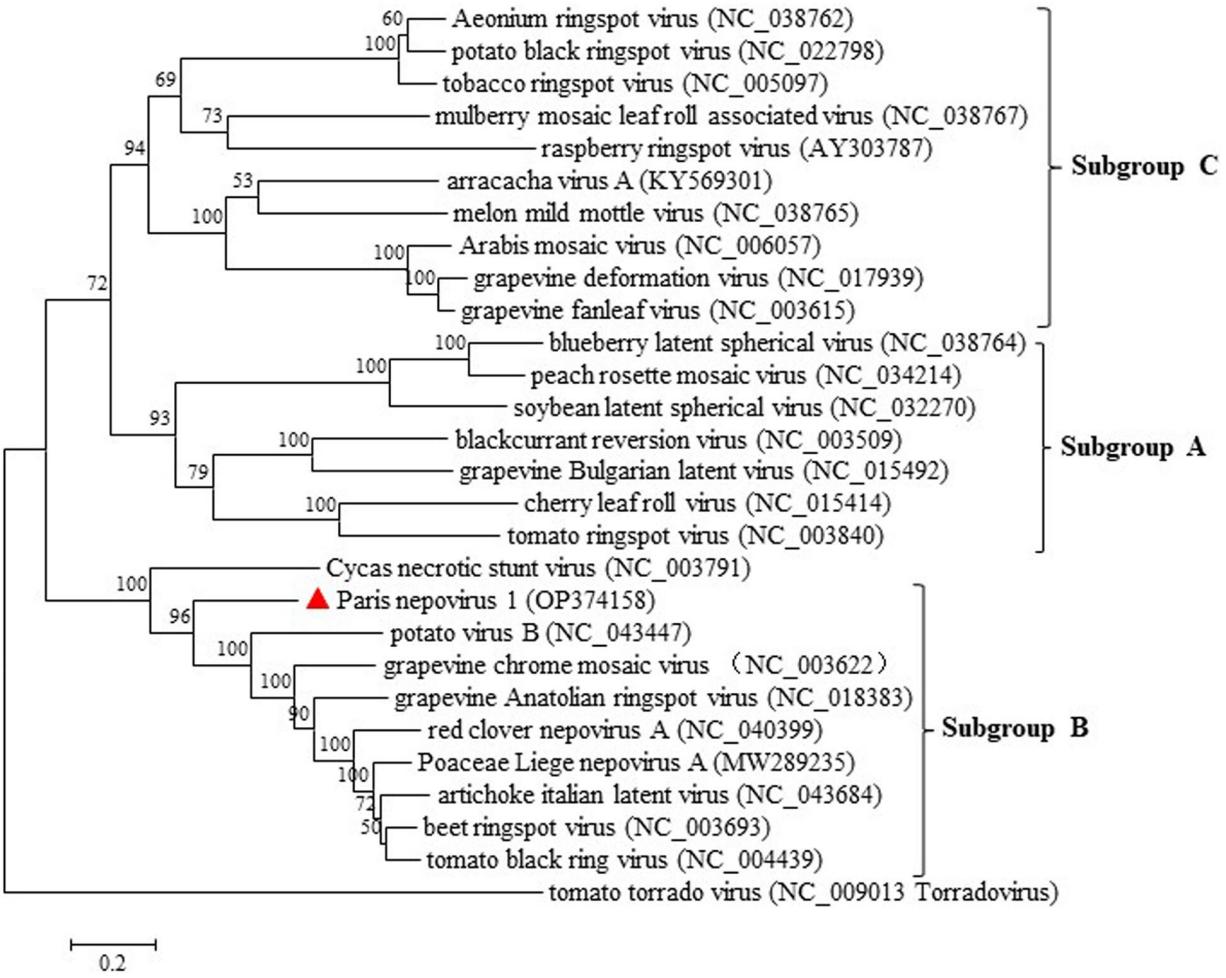
Maximum-likelihood tree based on the deduced polyprotein of ParNV-1 as well as those of the representative members in different subgroup of genus *Nepovirus*. Bootstrap analysis was applied using 1,000 bootstrap replicates. The scale bar representing a genetic distance of 0.2. Tomato torrado virus, a member of genus *Torradovirus*, was used as an outgroup. Solid triangle indicated the ParNV-1 isolate characterized in this study.

Pairwise comparisons of the conserved domains of ParNV-1 and those of the other nepoviruses showed that ParNV-1 and known members of subgroup B shared aa sequence identities of 49.9–63.7% at protease-polymerase (Pro-Pol) and 29.8% ~ 39.2% at CP, while the identities between ParNV-1 and members of other subgroups were only 28.8–36.9% at Pro-Pol and 21.0–23.6% at CP. These values are below the accepted species discrimination criteria (<80% aa identity at Pro-Pol and < 75% aa identities at CP) for the family *Secoviridae* (Thompson et al., 2017). A phylogenetic tree constructed using the polyprotein sequence of P1 of ParNV-1 and other known nepoviruses placed ParNV-1 into the subgroup B (Figure 5). These results confirm that Paris nepovirus 1 is a new member of the subgroup B in the genus *Nepovirus*.

### Sequence analysis of Paris isolate of LycMoV

**Figure 6:**
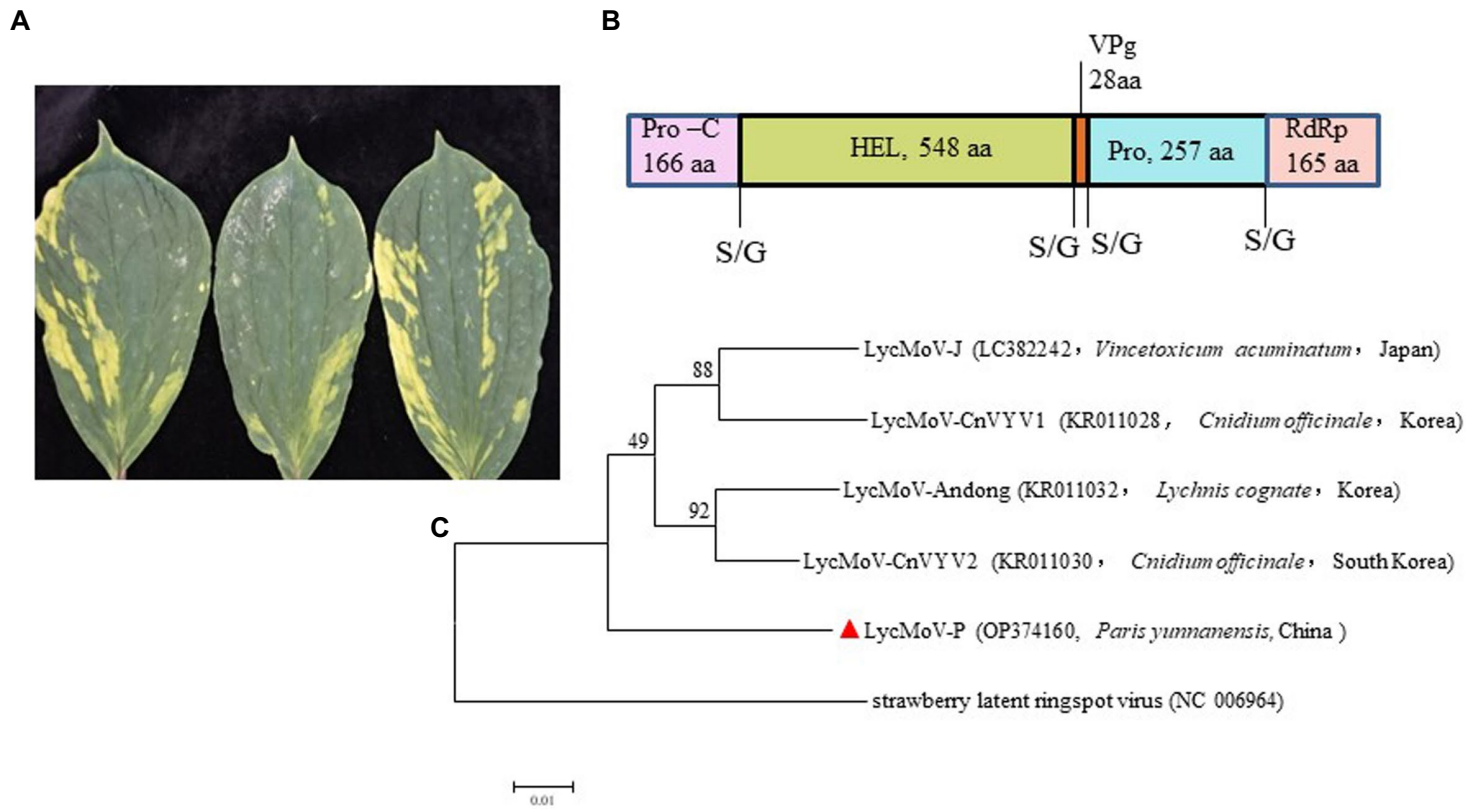
Symptoms, the structure for partial genomic sequence of RNA1, and phylogenetic tree of Lychnis mottle virus *P. yunnanensis* isolate (LycMoV-P): **(A)** Symptoms induced by co-infection of ParPV-3 + PMNV+LycMoV, **(B)** partial genomic structure of LycMoV-P RNA1, **(C)** Maximum-likelihood tree based on the partial aa sequence of LycMoV-P RNA1 and those of other isolates, and strawberry latent ringspot virus, another species in the genus *Stralarivirus*. Bootstrap analysis was applied using 1,000 bootstrap replicates. The scale bar representing a genetic distance of 0.01. Solid triangle indicated the LycMoV-P.

Lychnis mottle virus and strawberry latent ringspot virus are the only two members of the genus *Stralarivirus* in the family *Secoviridae* (Dullemans et al., 2020). In this study, a partial RNA1 sequence of 3,537 nt of the Paris isolate of LycMoV (LycMoV-P isolate; accession number: OP374160) was obtained by sequencing the amplicons from RT-PCR using virus-specific primers (Supplementary Table 5). The 1,164-aa polyprotein includes partial protease cofactor (Pro-C, 166 aa), helicase (HEL, 548 aa), genome-linked viral protein (VPg, 28 aa), cysteine protease (Pro, 257 aa) and partial sequence of RNA-dependent RNA polymerase (RdRp, 165 aa) (Figure 6B). Pairwise comparisons showed it shared aa sequence identities of 92.8–93.5% with other isolates of LycMoV at the same regions. A maximum likelihood tree based on the 1,164-aa polyprotein sequences grouped all five LycMoV isolates in a clade that is separated from strawberry latent ringspot virus (SLRSV) (Figure 6C). This is the first report of LycMoV in *P. yunnanensis* as well as in China.

### Sequence analyses of Paris isolate of HCRV

**Figure 7:**
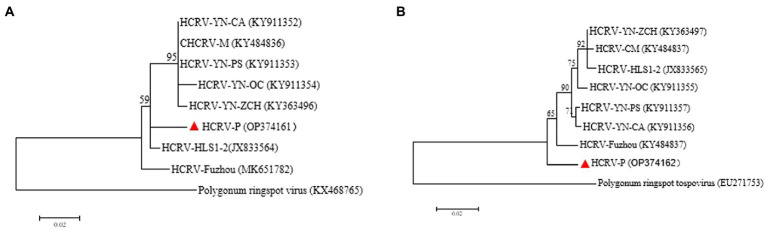
Phylogenetic tree of Hippeastrum chlorotic ringspot virus *P. yunnanensis* isolate (HCRV-P) and other isolates: **(A)** Maximum-likelihood trees were constructed based on the partial aa sequences of GPC, **(B)** ML trees were constructed based on the partial aa sequences of NP. Bootstrap analysis was applied using 1,000 bootstrap replicates. The scale bar representing a genetic distance of 0.2. Polygonum ringspot tospovirus, a species in the genus *Orthotospovirus*, was used as an outgroup. Solid triangle indicated the HCRV-P isolate characterized in this study.

Hippeastrum chlorotic ringspot virus is a member of the genus *Orthotospovirus* in the family *Tospoviridae*. The presence of HCRV in the diseased plants was verified by two overlapping amplicons of M RNA and an amplicon of S RNA (Supplementary Table 5). The 2,852-nt M fragment (HCRV-P isolate; accession number: OP374161) encodes partial glucoprotein precursor (GPC) of 325 aa residues (aa 152–1,128) on complementary sense, and the 697-nt S fragment (HCRV-P isolate; accession number: OP374162) codes for the N-terminus of nucleocapsid protein cistron (NP) of 217 aa residues (aa 1–217) on complementary sense. Pairwise comparisons showed the Paris isolate (P) and 7 other isolates of HCRV shared aa sequence identities of 95.6–97.1% at GPC and 95.9–97.7% at NP. Phylogenetic analyses using both GPC (Figure 7A) and NP (Figure 7B) places HCRV-P in a distinct clade separated from other clads, indicating HCRV-P is a distantly related to other HCRV isolates. HCRV is identified from *P. yunnanensis* for the first time.

### Detection of the eight viruses in *Paris yunnanensis*

**Figure 8:**
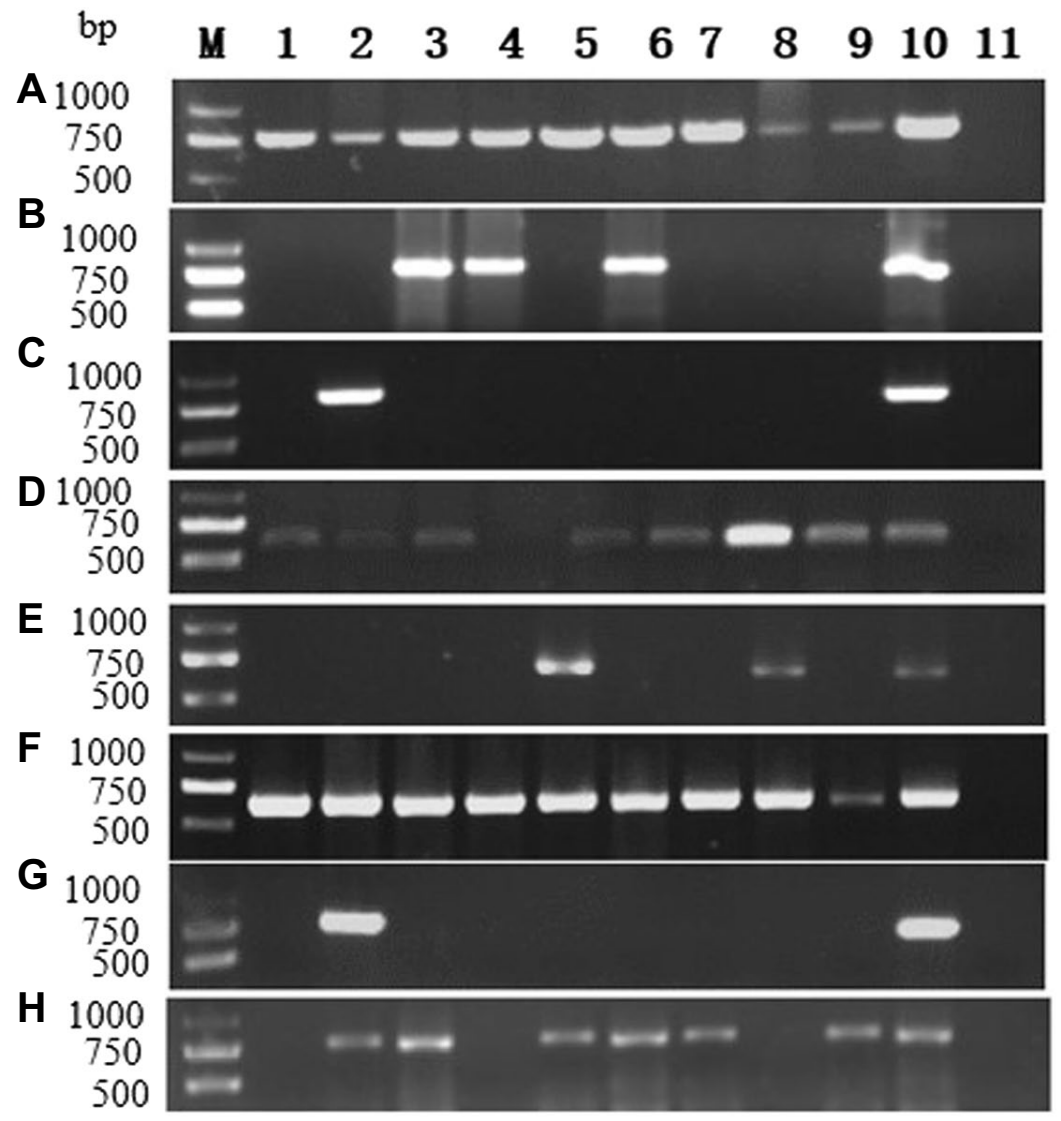
RT-PCR detection for viruses in diseased plants of *P. yunnanensis*: **(A)** ParPV-3, **(B)** ParPV-4, **(C)** ParNV-1, **(D)** HCRV, **(E)** LycMoV, **(F)** PMNV, **(G)** ParV1, **(H)** PMMoV. Lanes (M) DNA marker, (1–9) diseased samples, (10) positive control and (11) is negative control.

RT-PCR using virus-specific primers (Supplementary Table 1) were applied to determine the occurrences of the ten viruses in *P. yunnanensis*. Eight viruses including PMMoV (accession number: OP374164) and seven viruses [ParPV-3, ParPV-4, ParNV-1, HCRV, LycMoV, PMNV (accession number: OP374163) and ParV1 (accession number: OP374165)] identified in this study were detected (Figure 8) in single or mix infections from 65 of the 123 samples (52.8%) (Table 1). The average detection rates were 26.8% (33/123) of ParPV-3, 24.4% (30/123) of PMNV, 7.3% (9/123) of PMMoV, 4.1% (5/123) of HCRV, 3.3% (4/123) of ParPV-4, 1.6% (2/123) of LycMoV, 0.8% (1/123) of ParNV-1 and 0.8% (1/123) of ParV1 (Table 1). ParPV-3 and PMNV were predominant in the diseased plants, and 55 of the 65 positive samples (84.6%) were infected with at least one of these two viruses.

**Table 1:** Number of positive samples and infection rate of individual viruses in *Paris yunnanensis.*

			Viruses								
Region	Collected time	Number	ParPV-3	ParPV-4	ParNV-1	HCRV	PMMoV	PMNV	LycMoV	ParV1	# and % of positive samples
Mangshi	Aug-17	9	9	0	0	3	0	9	2	0	9
	Aug-19	9	0	0	0	0	8	1	0	0	8
Subtotal		18	9	0	0	3	8	10	2	0	17
Infection rate			50.00%	0	0	16.70%	44.40%	55.60%	11.10%	0	94.40%
Tengchong	Oct-19	36	13	0	0	0	1	12	0	0	25
Infection rate			36.10%	0	0	0	2.80%	33.30%	0	0	69.40%
Lijiang	Oct-19	54	4	3	1	2	0	8	0	1	16
Infection rate			7.40%	5.60%	1.90%	3.70%	0	14.80%	0	1.90%	29.60%
Lushui	April 2019	15	7	1	0	0	0	0	0	0	7
Infection rate			46.60%	6.70%	0	0	0	0	0	0	46.70%
Total		123	33	4	1	5	9	30	2	1	65
Detectable rate			26.80%	3.30%	0.80%	4.10%	7.30%	24.40%	1.60%	0.80%	52.80%

Mix infections occurred mainly among potyviruses or between a potyvirus and another virus. The infection rates were 5.6% (7/123) of ParPV-3 + PMNV, 2.4% (3/123) of ParPV-3 + PMNV+HCRV, 1.6% (2/123) of ParPV-3 + PMNV+LycMoV, 1.6% (2/123) of ParPV-3 + ParPV-4, 0.8% (1/123) of ParPV-4 + HCRV, PMNV + PMMoV and PMNV+ParV1 (Table 2), respectively.

**Table 2:** Co-infection of viruses in *P. yunnanensis.*

			Co-infection of viruses						
Region	Collected time	number	ParPV-3 + PMNV	ParPV-3 + ParPV-4	ParPV-4 + HCRV	PMNV+PMMoV	PMNV+ParV1	ParPV-3 +PMNV+HCRV	ParPV-3 +PMNV+LycMoV
Mangshi	Aug-17	9	6	0	0	0	0	3	2
	Aug-19	9	0	0	0	1	0	0	0
Tengchong	Oct-19	36	1	0	0	0	0	0	0
Lijiang	Oct-19	54	0	1	1	0	1	0	0
Lushui	Apr-19	15	0	1	0	0	0	0	0
Total		123	7	2	1	1	1	3	2
Detectable rate			5.60%	1.60%	0.80%	0.80%	0.80%	2.40%	1.60%

### Distribution of the eight viruses in the four counties

Single or mix infections and infection rates were different in the four surveyed counties. Five viruses, ParPV-3, PMNV, LycMoV, HCRV and PMMoV, were detected in 17 of the 18 samples (94.4%) from Mangshi (Table 1). The samples were positive for either one or 2–3 viruses (Tables 1, 2). PMNV, ParPV-3 and PMMoV were abundant, occurring in 55.6% (10), 50.0% (9) and 44.4% (8) samples. Different viruses were detected in the samples collected from Mangshi in 2017 and 2019. All 9 samples of 2017 tested positive for ParPV-3 and PMNV, whereas 7 of the 9 samples from 2019 were only positive for PMMoV. Only one plant of 2019 was doubly infected with PMMoV and PMNV. HCRV and LycMoV were only detected from the samples of 2017 in mix infections (Table 2).

Six viruses, ParPV-3, ParPV-4, ParNV-1, HCRV, PMNV and ParV1, were detected in 16 of the 54 samples from Lijiang (Table 1). Most of the positive plants (13/16, 81.3%) were infected with a single virus. PMNV was the most common virus, which was detected in 8 samples. Infection of the other five viruses was not common, with 1–4 samples tested positive. Mix infections occurred only in three samples (Table 2).

Fewer viruses were detected in Tengchong and Lushui. Three viruses were detected in 25 of the 36 samples (69.4%) from Tengchong, and majority of the infected plants (24) were infected with either ParPV-3 (13 samples, 36.1%) or PMNV (12 samples, 33.3%) (Table 1). Infection of PMMoV was rare, occurring only in one plant that was also infected with ParPV-3 and PMNV. Only ParPV-3 and ParPV-4 were detected in 7 of the 15 samples from Lushui, and ParPV-3 was predominant, detected in all the positive samples (Table 1). ParPV-4 was only detected from one of the positive samples as mix infection (Table 2).

ParPV-3 was the most widespread virus. It was detected in all the counties, with an overall infection rate of 26.8% (Table 1). Infection of PMNV was also common in Mangshi, Tengchong and Lijiang where 27.8% (30/108) samples were positive. ParPV-4, HCRV, PMMoV were detected in two counties, with ParPV-4 in Lijiang and Lushui, HCRV in Mangshi and Lijiang, and PMMoV in Mangshi and Tengchong. The infection rates were 5.6–6.7%, 3.7–16.7% and 2.8–44.4% (Table 1). The occurrences of LycMoV, ParNV-1 and ParV1 were limited to one county (Table 1). LycMoV was detected in Mangshi, while ParNV-1and ParV1 were found in Lijiang. The infection rates were 11.1% for LycMoV and 1.9% for both ParNV-1 and ParV1 (Table 1).

## Discussion

Plants of *P. yunnanensis* in their natural habits rarely express virus symptoms. However, virus-like diseases have become serious problems in the crop only after 40 years in cultivation. Several viruses have been previously found in the diseased *P. yunnanensis* (Dong et al., 2007; Lan et al., 2018; Wen et al., 2019; Chen et al., 2020, 2021; Yang et al., 2022). Aim to insight into pathogens of the viral diseases of various types in *P. yunnanensis*, morphology, virome analysis and virus surveys were conducted to study species, infection rates and distribution of the viruses in diseased plants in the four growth counties, Mangshi, Lijiang, Tengchong and Lushui, in this study. The viral diseases were found to be widespread in the fields of all the counties. Farmers noticed that the disease incidences gradually increased along with the growth years (personal communications). Virions of quasi-spherical and filamentous types were observed in the diseased samples (Figure 2). Virome analysis revealed the presence of seven viruses (ParPV-3, ParPV-4, ParNV-1, HCRV, PMNV, LycMoV and ParV1) in the symptomatic plants. PMMoV was detected by RT-PCR. Among them, ParPV-3, ParPV-4 and ParNV-1 are novel, HCRV and LycMoV are first found in *P. yunnanensis*, while PMNV, ParV1 and PMMoV are known viruses (Lan et al., 2018; Chen et al., 2020, 2021). The HTS and RT-PCR results confirmed the presence of multiple viruses in the diseased plants (Tables 1, 2).

Fifty of the 65 positive plants were infected with at least one potyvirus, and among them, ParPV-3 and PMNV were predominant in the fields. The four potyviruses of *P. yunnanensis* share nt sequence identities of 51.4–57.2% and belong to three subgroups, with ParPV-3 in the OrMV subgroup, ParPV-4 and ParV1 in the BYMV subgroup, and PMNV in the BCMV subgroup (Figure 4). Genetic divergences occur in these viruses, especially PMNV (Supplementary Table 6). The nt sequence identities among different variants obtained by Sanger sequencing (Lan et al., 2018) and HTS analysis in this study were 80.2–88.2%, indicating the presence of a high degree of divergence. Identification of multiple contigs of PMNV in both HTS samples suggests that the virus exists as ‘quasi’ species, which might be the results of low fidelity of its replicase and high pressure from this new host.

Members of the genus *Potyvirus* are usually transmitted by aphids (Atreya et al., 1991). Presence of the motifs involved in aphid transmission suggest the two new potyviruses might be transmitted by aphids that were abundant in the fields (personal communications). These viruses, therefore, could transmitted the viruses from crops or weeds grown in the same or nearby fields to *P. yunnanensis* and then spread by aphids, agricultural practices in fields or by contaminated seeds between different farms and regions. The relatively high infection rates of ParPV-3 and PMNV suggest that they might be transmitted efficiently by the insect vector/vectors. The aphid transmission and pathogenicity of these potyviruses need to be tested in the future.

PMMoV is an economically important pathogen of peppers (Capsicum spp.) worldwide (Kenyon et al., 2014). In natural conditions, PMMoV infects plants in Solanaceae, Chenopodiaceae, Amaranthaceae, Leguminosae and Cucurbitaceae through seeds, soil and agricultural practice. The virus is easily transmitted in fields by agricultural practice. Once a green pepper field has been contaminated with the virus, it is extremely difficult to eliminate the virus because it can survive up to 25 years on the remains of diseased plants (Ikegashira et al., 2014). PMMoV was first reported in *P. yunnanensis* in 2019 (Wen et al., 2019) and was mainly found in two farms at Mangshi (2019) and Tengchong in this study. We speculate that the virus crossed from infected peppers or weeds grown in nearby fields to *P. yunnanensis* in the two locations. Therefore, it is necessary to avoid growing peppers and other hosts of PMMoV around the *Paris* farms, clean the tools between use, and monitor the virus in sustainable cultivation of *P. yunnanensis*.

Nepoviruses cause a wide range of diseases on a variety of crops including both woody and herbaceous plants (Sanfaçon, 2008). Infections of the nepoviruses on many hosts are symptomless but some display symptoms such as necrotic, chlorotic rings or lines, mottling and flecking, vein necrosis, and plant stunting (Thompson et al., 2017). The ParNV-1 infected plants displayed chlorotic and necrotic spots, and pedicel austerity (Figure 1I) but its pathogenicity needs to be verified due to absence of inoculation data. Occurrence of ParNV-1 is rare, and only one plant was found to be infected in Lijiang. More samples from this location need to be tested for the virus in the future. Many nepoviruses are transmitted by nematodes and seed (Thompson et al., 2017), but evidence of both transmissions is not available for ParNV-1.

The orthotospovirus HCRV has a wide host range including *Hippeastrum rutilum*, *H. littoralis* (Dong et al., 2013), *Zephyranthes candida*, *Lactuca sativa*, *Emilia sonchifolia*, *Solanum lycopersicum*, *Phaseolus vulgaris*, *Vigna unguiculata*, *Tropaeolum majus*, *Cucumis sativus*, and *Cucurbita moschata* in family Amaryllidaceae, Asteraceae, Solanaceae, Leguminosae, Tropaeolaceae and Cucurbitaceae (Xu et al., 2013). In nature, HCRV is transmitted by thrips (*Taeniothrips eucharii*) and causes foliar necrotic and chlorotic ringspot symptoms on the infected plants. The symptoms caused by HCRV in *P. yunnanensis* were foliar mosaic and necrosis (Figure 1E) in single infection. However, biological assays are needed to verify its pathogenicity. The infection rates of the virus were low in the fields. To our knowledge, this is the first report of HCRV in *P. yunnanensis*.

Lychnis mottle virus is a member of the newly created genus *Stralarivirus*. it is second member of the family *Secoviridae* found in *P. yunnanensis* in this study. LycMoV was mainly reported in ornamental and medicinal plants, on which it induced irregular yellows and white spots on *Lychnis cognate* (Ran et al., 2015a), vein yellowing on *Cnidium officinale* (Ran et al., 2015b), mottle in *Vincetoxicum acuminatum* (Fujimoto et al., 2018) and asymptomatic in peonies (Shaffer et al., 2018). LycMoV was found to be associated with chlorosis in *P. yunnanensis* (Figure 6A), this association need to be further verified due to the mix infection with two potyviruses (Tables 1, 2). This is the first report of LycMoV in *P. yunnanensis* as well as in China. To better understand the virus and its pathogenicity in *P. yunnanensis*, the complete genomic sequences of the three RNA and the biological characters such as symptoms and host range of LycMoV-P should be further studied.

In this study, two large contigs represented nearly full-length genome of ParPV-3 (2 contigs from Mshi-CL) and ParPV-4 (2 contigs from Lji-CL) (Supplementary Table 6) were obtained from pooled samples by HTS, respectively. The two contig sequences of each virus were distinct from each other (Supplementary Table 6), suggesting that the presence of genetic divergence for both viruses in fields. One of the two variants was verified by RT-PCR cloning and Sanger sequencing. The nt sequence identities between HTS and the Sanger sequences were 99.7% between ParPV-3-YMS (OK073904) and contig-27-Mshi and 100% between ParPV-4-YLJ (OP374157) and contig-307-Lji, indicating the correct assembly in the HTS analysis.

The population structures of viruses infecting *P. yunnanensis* vary from region to region (Tables 1, 2). Thus, most, if not all, of these viruses infecting this newly cultivated crop might cross the species barriers, most likely from other unknown host species of medicinal herbs, ornamentals and vegetables grown in the same or nearby screenhouses and/or fields, to *P. yunnanensis* in separated incidences. The viruses were then spread from plants to plants by insect vectors and/or agricultural practice. The long growth period of the crop also facilitates the accumulation and spread of the viruses, causing rapid emergence of the diseases in the fields. However, it is not clear when, where and how these viruses were acquired but emergence of each of these viruses on this new crop can serve as a good model to study evolution of the viruses in the future (see Figures 7, 8).

## Data availability statement

The datasets presented in this study can be found in online repositories. The names of the repository/repositories and accession number(s) can be found below: https://www.ncbi.nlm.nih.gov/genbank/, OK073904; OP374157; OP374158; OP374159; OP374160; OP374161; OP374162; OP374163; OP374164; OP374165.

## Author contributions

FL and P-xL conceived and designed the research. PH, JY, and RG conducted the experiments. G-hZ and C-rL collected samples. RL analyzed the data from HTS. P-xL and RL analyzed the data and wrote the manuscript. X-jC and T-yW guided the experiments. All authors read and approved the manuscript.

## Funding

This study was funded by the Yunnan Academician Expert Workstation (202005AF150040) and the National Science Foundation of China (31660509 and 32260650).

## Conflict of interest

G-hZ was employed by Chinese Medicine Resources Co., Ltd.

The remaining authors declare that the research was conducted in the absence of any commercial or financial relationships that could be construed as a potential conflict of interest.

## Publisher’s note

All claims expressed in this article are solely those of the authors and do not necessarily represent those of their affiliated organizations, or those of the publisher, the editors and the reviewers. Any product that may be evaluated in this article, or claim that may be made by its manufacturer, is not guaranteed or endorsed by the publisher.

## Footnotes

^1^ https://www.ncbi.nlm.nih.gov/orffinder/

^2^ https://www.ebi.ac.uk/Tools/psa/emboss_needle/

^3^ https://www.ebi.ac.uk/Tools/msa/clustalo/

^4^https://www.ncbi.nlm.nih.gov/Structure/cdd/wrpsb.cgi.
