# Supplementary material for: High-throughput sequencing reveals the presence of novel and known viruses in diseased Paris yunnanensis
Source: Front Microbiol. 2022 Dec 14;13:1045750. doi: 10.3389/fmicb.2022.1045750 (PMC9795479; doi:10.3389/fmicb.2022.1045750)
Supplement: Supplementary file 1 [file Data_Sheet_1.docx]

Supplementary Table 1 Primers used for detection the viruses infecting *Paris .yunnanensis*

| Virus | Primer name* | Primer sequence (5’-3’) | Size (nt) | Tm (℃) | Note |
| --- | --- | --- | --- | --- | --- |
| ParPV-3 | ParPV-3DF | TGCGGACGATGGAACGATAG | 773 | 58 | This study |
|  | ParPV-3DR | CGAGGGAAAGGTGGGAAGTC |  |  |  |
| ParPV-4 | ParPV-4F1186 | TCTCATGGATGCTTCTCCGCG | 802 | 30 | This study |
|  | ParPV-4R1986 | CCGTAAGCTGGATGGACAACC |  |  |  |
| ParNV-1 | ParNV-1F2064 | TCACCGCAGAGAACAAGACC | 864 | 57 | This study |
|  | ParNV-1R3485 | GGCAACACAAAGGGATGCAG |  |  |  |
| PMNV | PMNVF8927 | TTGCATATGAATTGGAGGATACACAG | 579 | 55 | This study |
|  | PMNVR9506 | TATAACCACACTGAACTTAAAGAGAG |  |  |  |
|  |  |  |  |  |  |
| LycMoV | LycMoVDF | TTTCTACGTCTCTCAGTGTGGC | 703 | 56 | This study |
|  | LycMoVDR | ATCTTTCCTTACCTGCTTCACC |  |  |  |
|  |  |  |  |  |  |
| PPVX | PPVXDF | ATCTATGCAGTATCTAAGTCTAGTG | 852 | 55 | This study |
|  | PPVXDR | TGATTAGTGGTGTGACGCGGG |  |  |  |
|  |  |  |  |  |  |
| HCRV | HCRVDF | TTCTGGCCTAGAACTCTAGAGG | 540 | 56 | This study |
|  | HCRVDR | ACTACATTCTCTTCTAGGTTGGAG |  |  |  |
|  |  |  |  |  |  |
| ParV1 | ParV1DF | TTGATCCTGAAATTGATGCTGGTC | 799 | 55 | This study |
|  | ParV1DR | TCACATACGTACACCTAACATGC |  |  |  |
|  |  |  |  |  |  |
| PMMoV | PMMoVF | CAAACTTTATATTTCAGCACCTATGC | 728 | 55 | This study |
|  | PMMoVR | CGTTCGCAAATACACGTCAC |  |  |  |
|  |  |  |  |  |  |
| ChiVMV | ChiVMVdF | ATTCTTGAGTGGGATAGAGCTG | 1269 | 55 | Yang et al., 2021 |
|  | ChiVMVdR | TGGGAACCACACTGAGGAATATG |  |  |  |

* F: Forward primer; R: Reverse primer

Supplementary Table 2 Primers used for amplification of the complete nucleotide sequence of ParPV-3

| Primer name* | Primer sequence (5’-3’) | | Size (nt) | Tm (℃) | Note |
| --- | --- | --- | --- | --- | --- |
| ParPV-3-F40 | ATCTCTGAAATTGTCTCTTGATTCC | 1545 | | 55 | This study |
| ParPV-3-R1585 | AAACGTGCTAGTTGTAGTAGCTC |  |  |  |  |
| ParPV-3-F1442 | ACAGATTCAACAGCTGATTGGCC | 1554 | | 55 | This study |
| ParPV-3-R2996 | TGGACTCTTTGAAGCAACTAACC |  |  |  |  |
| ParPV-3-F2845 | AAGCGCATGCTGAAGCTCTC | 1616 | | 55 | This study |
| ParPV-3-R4461 | AGTATGTTATCGCCTCTGCTCAC |  |  |  |  |
| ParPV-3-F4436 | AAGTGGATTTGACACCCAGTATC | 1583 | | 55 | This study |
| ParPV-3-R5919 | ACATGATTTCCCTTGCGCTTGC |  |  |  |  |
| ParPV-3-F5804 | TTTCGCAACGCCAAGGATAGC | 1560 | | 55 | This study |
| ParPV-3-R7364 | TGTCGAGTATCCAGTACACATC |  |  |  |  |
| ParPV-3-F7239 | ATAGTAGACTCAACCGTGAGGC | 1995 | | 55 | This study |
| ParPV-3-R9234 | ATGTGAGCTTCTCTGGCACGC |  |  |  |  |
| ParPV-3-F9104 | AACACAGAGGGTAGGTATATGCC | ~600 | | 56 | For 3’-end |
| Via19 | GACCACGCGTATCGATGTCGACTTTTTTTTTTTTTTTV （V = A, C or G） |  |  |  | Lan et al., 2019 |
| Via18 | GACCACGCGTATCGATGTCGAC |  |  |  |  |
|  |  |  | |  |  |
| ParPV-3-R259 | ATTCGGGTCCTTCTTCGGCTCCGGGGC | ~300 | | 68 | For 5’ RACE |

* F: Forward primer; R: Reverse primer

Supplementary Table 3 Primers used for amplification of the complete nucleotide sequence of ParPV-4

| Primer name* | Primer sequence (5’-3’) | Size (nt) | Tm (℃) | Note |
| --- | --- | --- | --- | --- |
| ParPV-4-F121 | TCTTTGGTTCCATGACAAGCGAC | 1611 | 55 | This study |
| ParPV-4-R1669 | TAATTCGAGAAGAAGCGCTTGGC |  |  |  |
| ParPV-4-F1586 | AATGTGCGACAATCAACTCGAC | 1531 | 55 | This study |
| ParPV-4-R3188 | ACCTAACAACACGCTACACGC |  |  |  |
| ParPV-4-F3067 | TCACACACATTAGGCATAAGTGCAC | 1571 | 55 | This study |
| ParPV-4-R4607 | TCGTTTCTGGTATCTCATCATACGC |  |  |  |
| ParPV-4-F4449 | ATCGAAACTCATGGAACTGATACG | 1690 | 55 | This study |
| ParPV-4-R5977 | TTGTCGCTCAAGATGTACTCGG |  |  |  |
| ParPV-4-F5854 | ATTCGTTTGCCAGATATGTCGATC | 1553 | 55 | This study |
| ParPV-4-R7407 | TGTTTTCACGATCTCGGTCGGTC |  |  |  |
| ParPV-4-F7260 | AGAGAAGCTGGTTTCACACAGTG | 1501 | 55 | This study |
| ParPV-4-R8761 | AATTGCGCGTGAGTTGAACGCG |  |  |  |
| ParPV-4-F8590 | TGAACGTTGGAACACCTGGCAC | 799 | 55 | This study |
| ParPV-4-R9389 | AGAAGAGTGATGAGAAGAGGAGTC |  |  |  |
| ParPV-4-F9291 | TAGATGTATTGACTGGTGTGGTGC | ~600 | 58 | For 3’-end |
|  |  |  |  |  |
| ParPV-4-R232 | TCGCAAGTTTGAGTCGGTTAGATGTGAGAC | ~250 | 65 | For 5’ RACE |
| ParPV-4-R168 | TGTGCTGCATGGATTCGCCTTGC | ~200 | 60 |  |

* F: Forward primer; R: Reverse primer

Supplementary Table 4 Primers used for amplification of the complete nucleotide sequence of ParNV-1

| Primer name* | Primer sequence (5’-3’) | Size (nt) | Tm (℃) | Segment | Note |
| --- | --- | --- | --- | --- | --- |
| ParNV-1-1F39 | TTATGACCTGCGCCCTAGAGC | 11498 | 55 | RNA1 | This study |
| ParNV-1-1R1537 | TAAGGTGCAGAGACCAACTCCAC |  |  |  |  |
|  |  |  |  |  |  |
| ParNV-1-1F1370 | ATCAAGCACGAACTTGGAGTGGC | 1577 | 55 |  | This study |
| ParNV-1-1R2947 | TTGGTCCAGGTAGCACAGATGC |  |  |  |  |
|  |  |  |  |  |  |
| ParNV-1-1F2818 | TCACCGCAGAGAACAAGACC | 1599 | 55 |  | This study |
| ParNV-1-1R4417 | AATCGGCACGTTTGTTGGGAC |  |  |  |  |
|  |  |  |  |  |  |
| ParNV-1-1F4278 | ATGAGCCTGAGTTTGGTGAAGCG | 1650 | 55 |  | This study |
| ParNV-1-1R5928 | AGCATACCAGTCTGTTGTCCTG |  |  |  |  |
|  |  |  |  |  |  |
| ParNV-1-1F5746 | TGTTGTAGATGAACTCCTCCTC | 1024 | 55 |  | This study |
| ParNV-1-1R6770 | AGAAACCTGTGGACCCTGGTC |  |  |  |  |
|  |  |  |  |  |  |
| ParNV-1-1R102 | AGCCCTTAGGGACCATGGGTGTCTCGC | ~250 | 68 |  | For 5’RACE |
| ParNV-1-1R176 | TAGGGCCTTGGACACTTACGGGTGTC | ~350 | 65 |  |  |
|  |  |  |  |  |  |
| ParNV-1-1-F6676 | ATCCGCCTTTGAGGCTTGTTGGCTTGC | ~300 | 65 |  | For 3’-end |
|  |  |  |  |  |  |
| ParNV-1-2F70 | AAGACGAAGCTCTTACTCTCGC | 1578 | 55 | RNA2 | This study |
| ParNV-1-2R1648 | ATAAGCTGGTCCTCCACCTCC |  |  |  |  |
|  |  |  |  |  |  |
| ParNV-1-2F1488 | AAAGACACGCAAACGGCGTGC | 1636 | 55 |  | This study |
| ParNV-1-2R3124 | AACTCCTGGTCAATGGATGCAC |  |  |  |  |
|  |  |  |  |  |  |
| ParNV-1-2F2999 | TGATACCAATGTCAAGGTTGTGAG | 1481 | 55 |  | This study |
| ParNV-1-2R4480 | TATCCTAGCACCAACTTCAGAAGC |  |  |  |  |
|  |  |  |  |  |  |
| ParNV-1-2F2064 | ATCTGTGGTCCTCAATCTGCAC | 1421 | 55 |  | This study |
| ParNV-1-2R3485 | ATAACCACTAGACCCTTTCCTC |  |  |  |  |
|  |  |  |  |  |  |
| ParNV-1-2R106 | AGTGAGAATGAGATTGCGAGAGTAAGAGC | ~200 | 65 |  | For 5’ RACE |
| ParNV-1-2R177 | AGGCGCTATCCTTAACTTCCAAAGTGCG | ~300 | 65 |  |  |
|  |  |  |  |  |  |
| ParNV-1-2F4398 | TCCAACAGCCATAACAAGGACTTGGC | ~300 | 58 |  | For 3’-end |

* F: Forward primer; R: Reverse primer

Supplementary Table 5 Primers used for amplification partial sequence of LycMoV and HCRV

| Primer name* | Primer sequence (5’-3’) | Size (nt) | Tm (℃) | Virus | Segment |
| --- | --- | --- | --- | --- | --- |
| LycMoVF1861 | TCAATGCAGCTAGTTATGTTTGGG | 1523 | 55 | LycMV | RNA1 |
| LycMoVR3384 | AACCTGCGTATGTGGCGCATC |  |  |  |  |
| LycMoVF3385 | TAGGTACCGTTTTACTCTGCACC | 1514 | 55 |  |  |
| LycMoVR4738 | TGATGAGACTTCCAGAACCAGC |  |  |  |  |
| LycMoVF4596 | TTTCTACGTCTCTCAGTGTGGC | 704 | 55 |  |  |
| LycMoVR5299 | ATCTTTCCTTACCTGCTTCACC |  |  |  |  |
| HCRVMF1320 | TTCTGGCCTAGAACTCTAGAGG | 1489 | 55 | HCRV | M RNA |
| HCRVMR2660 | TCTCAACCTGCAGATGCACC |  |  |  |  |
| HCRVMF2555 | TTCATTTATGGCAAGGCAACCC | 1617 | 55 |  |  |
| HCRVMR4172 | AATCCTGTCTGATCCTGCCTC |  |  |  |  |
| HCRVSF1964 | TCAATGAAGCAGCACCAGTTGTC | 742 | 55 |  | S RNA |
| HCRVSR2706 | TTCAGCCAAGTAAGCAGCTTCC |  |  |  |  |

* F: Forward primer; R: Reverse primer

Supplementary Table 6 Viral contigs identified by high-throughput sequencing analysis

| Contig number^1^ | Length (bp) | Average coverage | Reference virus | Accession number | Sequence identities (aa)^2^ | Taxonomy (genus) | Sequence identities (nt)^3^ |
| --- | --- | --- | --- | --- | --- | --- | --- |
| 50-Lji | 6,902 | 2,782.34 | grapevine Anatolian ringspot virus segment RNA1 | NC_043532 | 55.2% | *Nepovirus* |  |
| 408-Lji | 4,886 | 2,242.70 | grapevine Anatolian ringspot virus segment RNA2 | NC_018383 | 39.3% |  |  |
| 25-Mshi | 9,504 | 20,425.25 | Kalanchoe mosaic virus | KY385304 | 51.7% | *Potyvirus* | 90.5% between them |
| 27-Mshi | 9,449 |  |  |  | 57.0% |  |  |
| 78-Lji | 9,523 | 383.52 | iris potyvirus A | MZ604653 | 56.7% | *Potyvirus* | 81.5% between them |
| 307-Lji | 9,497 |  |  |  | 57.1% |  |  |
| 15-Lji | 9,576 | 137,233.55 | Paris mosaic necrosis virus | NC_043532 | 88.1% | *Potyvirus* | 80.1-86.8% among variants |
| 16-Lji | 9,664 |  |  |  | 88.8% |  |  |
| 115-Lji | 9,621 |  |  |  | 90.5% |  |  |
| 25-Lji | 9,487 |  |  |  | 93.0% |  |  |
| 42-Mshi | 9,219 | 60,055.88 |  |  | 93.0% |  |  |
| 49-Mshi | 9,606 |  |  |  | 92.7% |  |  |
| 19-Mshi | 9,626 |  |  |  | 100% |  |  |
| 14678-Mshi | 3,559 | 25.31 | Lychnis mottle virus segment RNA1 | LC382242 | 95% | *Stralarivirus* |  |
| 49544-Mshi | 3,133 | 30.58 | Hippeastrum chlorotic ringspot virus segment M | KY363497 | 97% | *Orthotospovirus* |  |
| 7-Lji | 10,055 | 4,159.50 | Paris virus 1 | MN549985 | 89% | *Potyvirus* |  |

1. Number of viral contig identified by HTS analysis.

2. Amino acid sequence identity between the contig and reference sequences.

3. Nucleotide sequence identity between the contig sequences of the same virus.
